# Cellulose Nanocrystals (CNC)-Based Functional Materials for Supercapacitor Applications

**DOI:** 10.3390/nano12111828

**Published:** 2022-05-26

**Authors:** Arulppan Durairaj, Moorthy Maruthapandi, Arumugam Saravanan, John H. T. Luong, Aharon Gedanken

**Affiliations:** 1 Department of Chemistry, Bar-Ilan Institute for Nanotechnology and Advanced Materials, Bar-Ilan University, Ramat-Gan 52900, Israel; chemdraj@gmail.com (A.D.); lewismartin.jesus@gmail.com (M.M.); saran.bc94@gmail.com (A.S.); 2 School of Chemistry, University College Cork, T12 YN60 Cork, Ireland; luongprof@gmail.com

**Keywords:** cellulose nanocrystal, surface functionalization, conductive electrodes, energy storage, supercapacitors

## Abstract

The growth of industrialization and the population has increased the usage of fossil fuels, resulting in the emission of large amounts of CO_2._ This serious environmental issue can be abated by using sustainable and environmentally friendly materials with promising novel and superior performance as an alternative to petroleum-based plastics. Emerging nanomaterials derived from abundant natural resources have received considerable attention as candidates to replace petroleum-based synthetic polymers. As renewable materials from biomass, cellulose nanocrystals (CNCs) nanomaterials exhibit unique physicochemical properties, low cost, biocompatibility and biodegradability. Among a plethora of applications, CNCs have become proven nanomaterials for energy applications encompassing energy storage devices and supercapacitors. This review highlights the recent research contribution on novel CNC-conductive materials and CNCs-based nanocomposites, focusing on their synthesis, surface functionalization and potential applications as supercapacitors (SCs). The synthesis of CNCs encompasses various pretreatment steps including acid hydrolysis, mechanical exfoliation and enzymatic and combination processes from renewable carbon sources. For the widespread applications of CNCs, their derivatives such as carboxylated CNCs, aldehyde-CNCs, hydride-CNCs and sulfonated CNC-based materials are more pertinent. The potential applications of CNCs-conductive hybrid composites as SCs, critical technical issues and the future feasibility of this endeavor are highlighted. Discussion is also extended to the transformation of renewable and low-attractive CNCs to conductive nanocomposites using green approaches. This review also addresses the key scientific achievements and industrial uses of nanoscale materials and composites for energy conversion and storage applications.

## 1. Introduction

Environmental safety and renewable sources are two prerequisites for energy conversion and storage fields. However, the extensive usage of petroleum-based synthetic polymers for energy conversion applications results in severe environmental issues [1,2]. For the last two decades, the widespread utilization of synthetic polymers has instigated critical issues through an accumulation of plastic wastes, the depletion of fossil fuels and global climate change. Owing to diversified industrialization, the global CO_2_ emissions from petroleum-based fossil fuels increase daily, and this trend will continue in the future [3]. To mitigate these environmental issues, sustainable and renewable biopolymers can be an alternative source for the development of efficient viable products [4]. Natural biomass and biowaste including lignocellulose are abundant and renewable sources for the production of value-added products without any harmful effect on the ecosystem [5]. The most abundant cellulose has many attractive features such as nontoxicity, renewability, biodegradability, low cost and colloidal stability [6]. The cellulosic natural biomass contains various other components including waxes, lignin, pectin and inorganic nitrogenous salts [7]. A pretreatment process is necessary to fractionate nanocellulose from cellulose resources such as plants, woods, algae, tunicate and bacteria with a polysaccharide structure [8,9,10]. It comes up as a promising, eco-friendly and sustainable candidate with a unique structure and remarkable properties for further structural modifications. Nanocellulose has been successfully investigated for its various applications in energy, packaging, paper making, sensors, cosmetics, coating and environmental remediation areas [11,12]. Nanocellulose [13] is termed to depict two emerging dominant materials: cellulose nanocrystals (CNCs) or nanocrystalline cellulose (NCCs) [14] and cellulose nanofiber (CNF) [15]. Nanocellulose can be prepared from cellulose, a linear chain of β-D-glucopyranose units, via β-1, 4 glycosidic bonds with intensive intra/intermolecular hydrogen bonding. The preparation of CNCs is obtained by traditional acid hydrolysis [16], mechanical disintegration [17], chemical oxidation and hydrolysis by ammonium persulfate [14].

A cellulose nanocrystal resembles a needle-shaped structure; it is 100–500 nm in length and 1–50 nm in diameter [14]. CNCs are the most ideal material, with attractive features including good mechanical strength, colloidal stability, biodegradability and very low cytotoxicity [18,19]. CNCs have gained considerable attention because of their attractive cost and diversified industrial applications and bioapplications [20,21,22,23]. In this context, CNCs have been advocated for supercapacitor applications and lithium-ion batteries [24,25,26]. One of the three energy storage technologies [26], along with mechanical energy storage and biological energy storage, electrochemical energy storage systems comprise lithium batteries, lithium-sulfur batteries and supercapacitors [27,28]. SCs have gained extensive consideration due to their charge-discharge ability, high cycle stability, high energy conversion and environmentally friendly technologies [29,30,31]. SCs have a high specific capacity and high energy density in contrast with traditional capacitors. Compared with secondary batteries, they are generating less pollution and fewer chemicals. They also have an extended life cycle, a broad operating temperature and a fast charge-discharging capability [32,33]. Currently, SCs are widely used for energy vehicles, wearable electronic products, etc. [34,35].

SC devices consist of electrodes, electrolytes, current collectors and a diaphragm. As the backbone of the supercapacitor, an electrode is an important tool for the effective conversion and storage of electrochemical energy [36,37]. Supercapacitors are energy-stored energy devices, which are based on three major principles: EDLC (electrical double layer capacitance), pseudocapacitance and asymmetric supercapacitance. EDLC is attained by the separation of charges between the Helmholtz double-layer and the diffusion layer at the electrolyte interface [29]. Graphene oxide, activated carbon, reduced graphene oxide and heteroatom doped carbon have a high power density and recyclability. Unlike EDLC, pseudocapacitors are formed through the reduction–oxidation reactions resulting from chemical transformations [30]. The major principle involved in pseudocapacitors is the transfer of electron charge between the electrode and electrolyte through reduction–oxidation reactions, electrosorption and intercalation. Pseudocapacitor materials are generally made up of metal oxides such as RuO_2_, NiO, MnO_2_, Co_3_O_4_, conducting polymers and metal sulfides. Pseudocapacitors exhibit a high specific capacitance, a high energy density and a high power density; however, a limited lifetime and reduced cell voltage are their two major disadvantages [31]. Asymmetric supercapacitors (ASCs) or hybrid supercapacitors are fabricated by combining two different electrode materials with special properties for increasing the cell voltage and power density of supercapacitor materials. Supercapacitors exhibit fast charge-discharge, an extended cycle lifetime, a high efficiency for the charge and discharge cycle, eco-friendliness, etc. [37]. Therefore, the selection of novel electrode materials is one of the main tasks for the fabrication of high-performance SCs. To date, carbon-based materials, transition metals, graphene-based materials and conductive materials are commonly used electrode materials for SCs [38,39,40]. These nanomaterials were successfully applied as supercapacitor electrodes for many energy applications including for automobiles and electronic gadgets such as watches, laptops, etc. [41,42]. However, the existing SC electrode system often fails to construct efficient electrochemical energy charge-discharge behavior [43,44]. Thus, research endeavors have focused on the development of green carbon-based nanomaterials with highly efficient capacitor electrodes. This strategy is considered a good pathway to reduce the agro-industrial waste and generate revenue for supply chains—a significant shift toward the circular economy [45]. Porous green-based carbon materials with a high surface area and bounteous heteroatoms can serve as electrode materials with boosted electrochemical activities [46,47]. In particular, CNCs can be the ideal material for SCs due to their high crystalline nature, surface functionalization ability and abundant nature [48,49]. However, critical findings on the collective evaluation of CNC-based electrode materials in the field of SCs remain a subject of future endeavors. 

**Figure 1:**
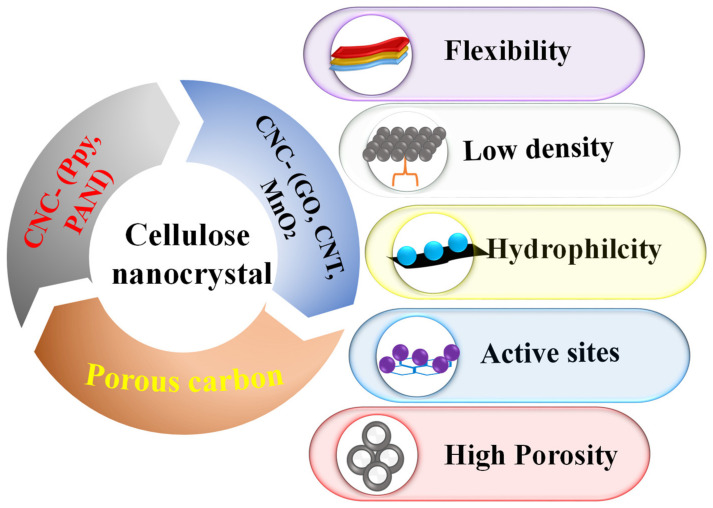
Schematic illustration of the hybrid cellulose nanocrystal and its properties.

This comprehensive review highlights the research findings of novel CNCs designed for energy storage applications. It elaborates on the significance of renewable and innocuous CNCs as low-cost natural resources and essential features in supercapacitor applications. Different synthesized methods of CNCs will be discussed including the pretreatment of cellulose sources, processing parameters and surface functionalization. The pretreatment process plays a vital role in eliminating the lignin and other unwanted impurities from the cellulose and in increasing the crystallinity of the CNCs. For the enhanced dispersion of CNC in hydrophilic and hydrophobic matrixes, selective surface modification techniques including chemical modification and enzymatic modification are discussed. CNC electrodes in SCs consist of a conductive polymer/CNC, porous carbon derived from CNCs, hybrid CNC electrodes as well as corresponding doped materials (Figure 1). This in-depth review highlights the key challenges and prospects of CNC-based SC electrodes.

## 2. Cellulose Nanocrystals (CNCs): Structure and Properties

**Figure 2:**
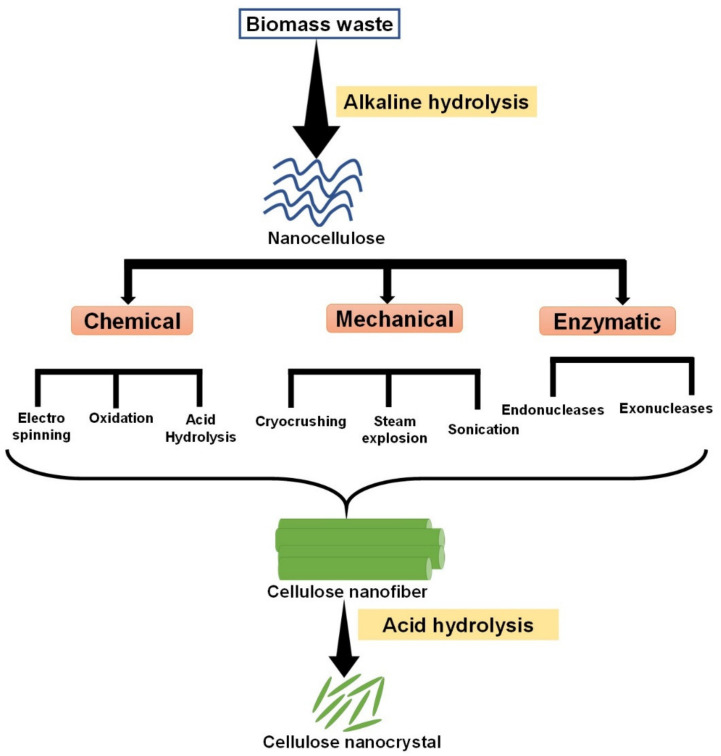
Different schemes for making cellulose nanocrystals (CNCs).

CNCs have gained enormous attraction due to their novel nanostructure and highly crystalline nature [14,21]. These emerging materials enable the development of highly efficient nanocomposites with unique properties. Besides renewable and abundant plants and woods, [50,51], CNCs are often derived from cotton, bacterial tunicate, microcrystalline cellulose (MCC) and biowaste materials [14,21,52,53]. CNCs are prepared from cellulose under controlled hydrolysis conditions, resulting in a stable CNC suspension. Cellulose has two distinct components: amorphous and crystalline [14,21]. When it is subjected to conventional acid hydrolysis [54] or chemical oxidation and hydrolysis [14], the amorphous part is removed from parental cellulose, resulting in a shorter CNC with high crystallinity. Among the various acids and oxidants, the amorphous domain of cellulose can be easily removed by concentrated sulfuric acid [54]. Compared to cellulose, CNCs are shorter *β*(1–4) connected chains of anhydrous-glucopyranose units (AGU)—a short-rod-like shape or whisker shape (diameter: 2–20 nm and length: 100–500 nm) with higher crystallinity [14,55,56]. Like cellulose, all the hydroxyls are placed in every equatorial position in the chair conformation, which allows for the stable nature of CNCs. The equatorial hydroxyl groups are also stabilized by intramolecular hydrogen bonding, providing enhanced mechanical strength. The CNC properties can be altered by surface modification with suitable materials [14,57] and gold nanoparticles for specific applications [58]. Some other important properties of CNCs are (i) their high mechanical strength, (ii) the creation of 3D nanostructured nanomaterials through their intermolecular interactions and (iii) their tunable surface modification [14,56,59]. The schematic preparation of CNCs is portrayed in Figure 2.

The development of surface-modified CNCs offers a plethora of potential applications encompassing energy, packaging, special papers, paints, building materials, aerospace, biomedical materials, pharma industries, cosmetics, the electronic and automotive industries, etc. [60,61,62].

## 3. Pretreatment Process

As an attractive source of the production of nanocrystalline cellulose, natural biomass contains various components such as cellulose, lignin, hemicelluloses, etc. [63]. Various types of pretreatment processes, shown in Table 1, must be carried out for the removal of noncellulosic components [64]. The traditional pretreatment process includes alkaline treatment and acid chloride treatment to remove both hemicellulose and lignin from renewable biomass. These methods are often referred to as “delignification”, as they effectively eliminate lignin and other components from the biomass, resulting in pure cellulose. The acid hydrolysis of cellulose from different sources, e.g., cotton and wood, has been frequently used to prepare CNCs [65]. Besides two strong acids, sulfuric and hydrochloric acids, phosphoric acid and hydrobromic acids are mostly used to prepare rod-like CNCs from cellulose feedstocks. Strong acids are polluted, corrosive and adversely affect the heat resistant nature of CNCs [14,56].

Greener methods have emerged to minimize the usage of sulfuric acid for the synthesis of CNCs en masse. Of note is the preparation of CNCs from palm oil through the TCF (total chlorine-free) method; however, the resulting CNCs are subjected to notable degradation during this treatment [66]. Two ionic liquids, 1-propyl-3-methylimidazolium chloride and 1-ethyl-3-methylimidazolium chloride, are used to prepare CNCs (average diameter of 20 nm) from Avicel [67], the commercial Sigma-Aldrich microcrystalline cellulose (MCC). Avicel is prepared by the acid hydrolysis of specialty wood pulp and purified and partially depolymerized alpha-cellulose. The yield of CNC from biomass can be improved through various strategies such as solution plasma processing technology [68], ultrasound-assisted enzymatic hydrolysis [69], microwave-assisted acid hydrolysis [70] and sonication-assisted TEMPO (C_9_H_18_NO, 2,2,6,6-tetramethylpiperidine 1-oxyl, 2,2,6,6-tetramethyl-1-piperidinyloxy) oxidation [71]. One-pot TEMPO-periodate oxidation reactions form highly-carboxylated CNCs [72]; however, TEMPO is very expensive, which is a deterrent factor for the mass preparation of CNCs. In this context, the use of ammonium persulfate (APS) [14,56], a patented technology [73], offers a low-cost chemical for the synthesis of carboxylated CNCs. 

CNCs can also be prepared from bamboo pulp fibers using a weak acid, e.g., maleic acid, together with a ball mill pretreatment process. The ball milling mechanical force decomposes the bamboo fiber and promotes acid hydrolysis effectively. The yield of ball-milled pretreated CNC is 10.55–24.50% higher than that of the normal acid hydrolysis without ball milling mechanical forces [74]. Ultrasonication can be used to minimize the amount of sulfuric acid in the preparation of CNCs from MCC [75]. Ultrasonicated CNCs exhibit higher thermal stability compared to their counterparts obtained by concentrated acid hydrolysis. Perhaps ultrasonicated pretreatment is one of the facile techniques for making CNCs from biomass feedstocks [75]. Microwave pretreatment assisting with alkali treatment is another green approach for the synthesis of CNCs from seaweed. This technique eliminates the wax from seaweed fibers and reduces the alkali effect with a short heating time [76]. Of interest is the design of a deep eutectic solvent method to prepare high crystalline CNCs without any chemical functionalization. The deep eutectic solvent method may be an environmentally friendly, renewable, biodegradable and non-toxic technique to prepare CNCs in the future [77]. The environmentally friendly pretreated steps are critical in the large-scale production of CNCs for industrial applications. 

## 4. Surface Modification of CNCs

**Figure 3:**
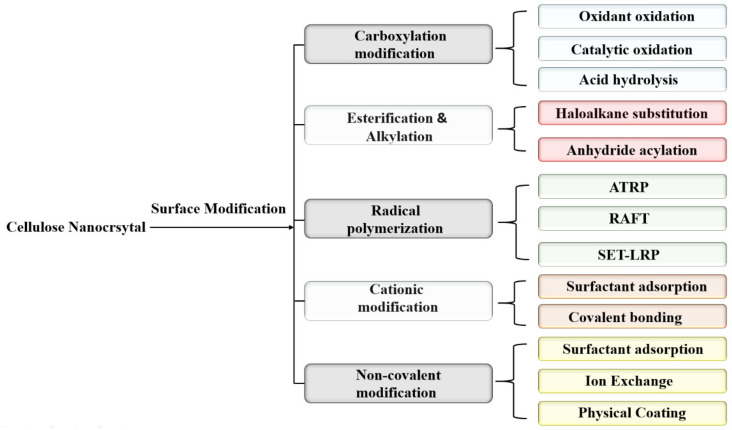
Surface functionalization of CNCs by different routes.

**Figure 4:**
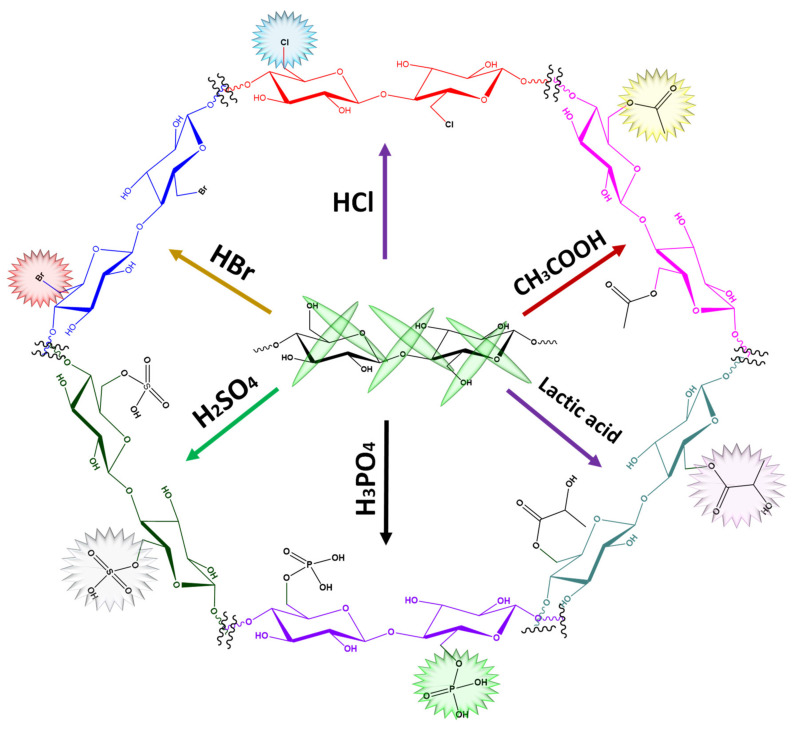
Common surface functionalization techniques of CNCs.

With high crystallinity, CNCs are not reactive, and the hydrophilic behavior of CNCs, particularly sulfonated and carboxylated CNCs, impedes their dispersion in hydrophobic matrices. Therefore, the incorporation of pertinent groups on CNCs is required to increase their physiochemical properties and endow innovative applications. The surface functionalization of CNCs fulfills the recent challenging needs in developing applications such as wastewater treatment, polymer composites, barrier films, textiles, energy and biomedical applications [78,79]. In brief, the CNC surface can be modified with covalent methods (acetylation, amidation, benzoylation, silanization, esterification, isocyanation and polymer grafting) and non-covalent methods (the introduction of polymers, surfactants and compatibilizing agents). Among the various routes available for the surface functionalization of CNCs [80], the reaction must be performed under strictly controlled conditions to preserve the distinct crystalline behavior of CNCs. Functionalized CNCs from innovative techniques (Figure 3 and Figure 4) will foster advanced and novel applications of CNCs.

## 5. Oxidation of Cellulose Nanocrystals (CNCs)

A commercial oxidizing reagent (TEMPO) selectively converts some hydroxyl functional groups to carboxyl functional groups on the cellulose moiety at the C-6 position. The oxidized CNCs with carboxyl groups from the HCl hydrolysis pretreated cellulose fibers show a better dispersion in the aqueous medium [14,81]. The TEMPO-oxidized CNCs exhibit the same morphology and excellent dispersion in water after the incorporation of the carboxyl group [82,83]. Other oxidizing reagents such as ammonium persulfate [14,56] and chlorite-periodate are successfully used for CNC surface modification through the oxidation process, as mentioned earlier. APS can defibrillate and remove the amorphous domain of cellulose effectively, resulting in uniform high-crystalline carboxylated CNCs [14,84]. 

## 6. Acetylation of CNCs

Acetylated CNCs can be obtained by reacting CNCs with acetic anhydride under the dimethylformamide (DMF) solvent medium [85]. After heating at 105 °C for 24 h and thorough washing with water/methanol/acetone to remove unreacted CNCs, the resulting CNCs are easily dispersed on the nonpolar polymeric matrix by the reduction of hydrogen bonds. The combined acid hydrolysis and acetylation reduce the crystallinity and adversely affect the morphology of the acetylated CNCs. CNCs extracted from cotton fibers can also be acetylated by vinyl acetate, and this one-step pathway is carried out in dimethylformamide (DMF) at 95 °C [86].

## 7. Sulfonation of CNC

The acid hydrolysis of cellulose by sulfuric acid produces CNCs with some sulfate moieties [87], which increase their dispersion in aqueous media. Even with the optimized procedure, the resulting sulfate functional groups on the CNCs are still limited, and this is a subject of future endeavors for the preparation of CNCs with sufficient sulfate groups [9]. 

## 8. Silylation of CNCs

Hydrophobic alkyl groups such as chlorosilanes and alkoxysilanes can be introduced to CNCs by silylation with silane coupling agents. Normally, silane coupling agents are used in composites for adhesive applications. They contain several functional groups, which easily couple with different nanomaterials through covalent linkage or van der Waals interaction [88]. Silanized CNCs become more hydrophobic, with improved dispersion in organic solvents compared to pristine CNCs. The coupling of the silane functional groups on CNCs starts with hydrolysis, which introduces alkoxyl groups in acidic, basic and/or neutral pH conditions to form the silanol (Si–O–Si). The formation of silanol is evidence of the elimination of hydroxyl or water from the CNCs [89]. Silane coupling agents can be used to develop silylated CNCs. During the silanization, hydrolysis and condensation reactions occur on the CNC surface. The combined esterification and depolymerization process leads to the creation of Si-O-C bonds [90].

## 9. Carbamation

CNCs subjected to isocyanates such as 2,4-diisocyanatotoluene, 3,5-dimethylphenyl isocyanate, n-octadecyl isocyanates (OI), hexamethylene diisocyanate and methylene diphenyl diisocyanates form carbamated CNCs with enhanced hydrophobicity [91,92]. For example, isocyanate modified CNCs can be prepared using isophorone diisocyanate under dimethylsulfoxide (DMSO) at 60 °C [93]. Modified CNCs can be dispersed in a polyurethane matrix after the incorporation of IDPI (isophorone diisocyanate). Carbamated CNCs are prepared by the treatment of pristine CNCs with 2,4-toluene diisocyanate in the presence of triethylamine. The *p*-NCO and *o*-NCO functional groups replace the OH groups of CNCs, which have a higher reactivity than pristine CNCs [94].

## 10. CNCs: Key to Developing Sustainable Electrochemical Energy Material

**Figure 5:**
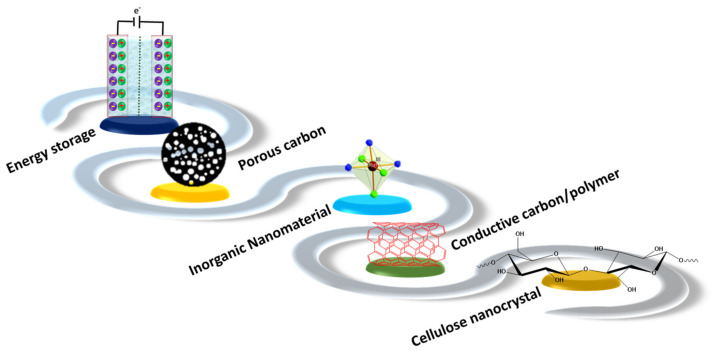
Schematic representation of CNC building blocks and active materials for supercapacitor devices.

**Figure 6:**
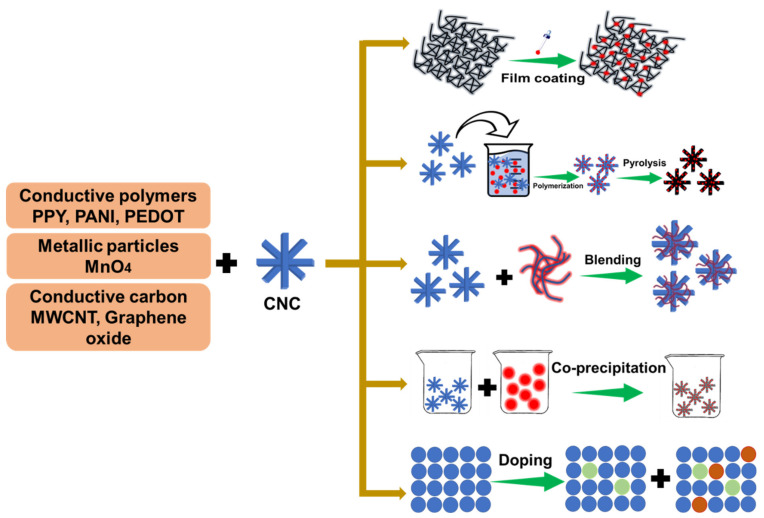
Various methodologies to develop novel conductive cellulose nanocrystals (CNCs).

Upcoming sustainable development focuses on utilizing renewable materials for the development of value-added, environmentally friendly products. CNCs derived from renewable biomass have been advocated for diverse applications in the paper industries, energy storage, remediation, optoelectronic fields, etc. Of interest are the potential uses of CNCs in SCs, Li-ion batteries, solar cells and electrochemical energy storage devices [95,96]. However, CNCs still require enormous surface conductivity to become ideal materials for important energy applications. In this context, the CNC surface can be modified with conductive polymers, MWCNT (multiwalled carbon nanotube), graphene nanosheets and some inorganic nanomaterials, as shown in Figure 5. This endeavor aims to develop highly efficient energy storage systems containing CNCs. In this context, low-cost and flexible CNCs have shown great promise as effective electrode materials [97]. Two major approaches have been applied to develop CNC-based SCs such as coating the conductive material on the surface of CNCs and mixing conductive materials with CNCs via in situ polymerization and blending. The in situ polymerization strategy is followed to develop a conductive polymer on the CNC matrix from the repetitive building block of monomers to build CNC-conductive polymer composites. The blending approach is used to combine different active nanomaterials with CNCs to make effective hybrid composites via synergistic effects [98]. In addition, co-precipitation and doping methods have been applied to prepare CNC-conductive electrodes. The facile preparation of conductive cellulose nanocrystal electrodes is depicted in Figure 6. 

## 11. Conductive Polymer CNCs

Two traditional and popular conjugated polymers, e.g., polypyrrole (PPy) and polyaniline (PANI), are conducting macromolecules with their backbone comprising alternating single and multiple bonds [99]. With their unique properties, conjugated polymers are widely studied as electrochemical capacitors, solar cells, OLED, fuel cells and sensors. However, the solubility of conjugated polymers is very limited [100], and this setback can be overcome by the preparation of nanocomposites between conducting polymers and CNCs. In principle, the conjugated polymers such as PPy, PANI, polyacetylene, polythiophene, poly(*p*-phenylene vinylene) and their derivatives are used to make CNC-conductive polymer electrodes. As an example, the fabrication of a poly(3,4-ethylenedioxythiophene) (PEDOT)/CNC conductive electrode can be realized by a simple electrochemical polymerization technique [101]. The formation of PEDOT on CNC depends on the effect of the electrochemical polymerization potential, including the applied potential, deposition time and concentration of the precursors. The PEDOT/CNC electrode exhibits a specific capacity capacitance (C_s_) of 117.02 Fg^−1^ at 100 mVs^−1^, with an E_s_ (energy density) and P_s_ (power density) of 11.44 Whkg^−1^ and 99.85 Wkg^−1^, respectively, at a 0.2 Ag^−1^ current density. The combination of PEDOT and CNCs shows a lower charge transfer resistance (R_ct_  =  0.53 Ω), with a retention capacitance of 86% after 1000 cycles. The presence of SO_3_^−^ groups being counter ions to the positively charged PEDOT on the CNC surface serves as a supportive framework in the formation of PEDOT/CNC. The functional groups on modified CNCs also prevent the severe structural changes of the electrode during electrochemical storage applications [101]. 

**Figure 7:**
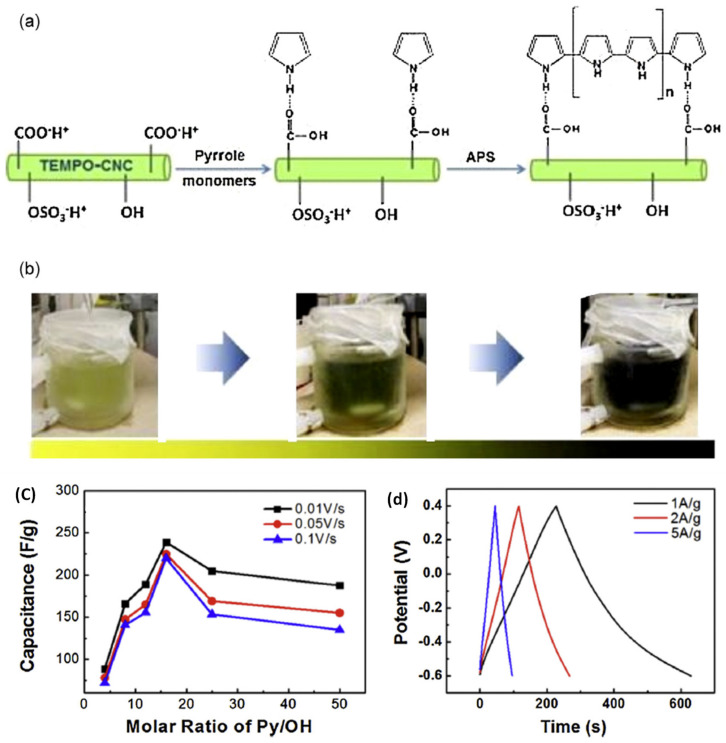
(**a**,**b**) In situ polymerization synthesis of CNC/PPy, (**c**) Effect of PPy on the capacitance of CNC/PPy, (**d**) Charge and discharge test of CNC/PPy at 1 Ag^−1^, 2 Ag^−1^ and 5 Ag^−1^ (charge current). Adapted with permission from [102]. Copyright 2014 Elsevier.

A novel, simple and scalable conductive PPy/CNC electrode for supercapacitor applications is attained via the in situ chemical polymerization method **[102]**. CNCs treated with TEMPO possess carboxylic acids on their CNC surface (Figure 7). The pyrrole monomer is then deposited and polymerized on the surface of carboxylated CNCs to construct a PPy/CNC electrode with 248 Fg^−1^ (C_s_) at 10 mVs^−1^, compared to the 90 Fg^−1^ of a pristine PPy electrode. Hence, carboxylated CNCs play a vital role in the charge-discharge mechanism during electrochemical energy storage applications. The extraordinary specific capacitance behavior of PPy/CNCs can be attributed to the favorable polymerization of PPy on carboxylated CNCs, the strong interaction between CNCs and PPy, the porous nature of PPy/CNC and the low density of CNCs [102]. Another attempt is the fabrication of a porous CNC/PPy electrode using the electrochemical co-deposition method [103]. Again, CNCs prepared from cotton by acid hydrolysis are subjected to TEMPO to form carboxylated CNCs. The CNC/PPy electrode is developed by the electrodeposition of pyrrole and carboxylated CNCs. The Cl^−^ ions from the PPy are incorporated to balance the positively charged CNC skeleton. The CNC/PPy and PPy electrodes exhibit 336 Fg^−1^ and 258 Fg^−1^, respectively, and the former’s high C_s_ is attributed to the high porous morphology in the composite. In addition, the PPy favors ion transfer between the CNCs and electrolyte interface. The CNC/PPy electrodes are also compared with the PPy/carbon nanotube electrode prepared by similar electrodeposition. The CNC/PPy nanocomposites reveal comparable specific capacitance and good stability compared to the CNT/PPy electrode [103].

Similarly, the CNC/PANI and CNC/PEDOT composites have been proven as electrochemical supercapacitors [104]. CNCs are prepared from a cotton source and are oxidized by TEMPO. The CNC/PANI and CNC/PEDOT electrodes are then prepared by an electrodeposition process consisting of CNCs, HCl, aniline and 3,4-ethylenedioxythiophene. The electrodepositing polymer films exhibit a porous morphology, which facilitates the movement of ions between the electrode and electrolyte interface. The C_s_ of the CNC/PANI, PANI, CNC/PEDOT and PEDOT is 488 Fg^−1^, 358 Fg^−1^, 69 Fg^−1^ and 58 Fg^−1^, respectively. The CNC/PANI electrodes show higher C_s_ than the CNC/PEDOT due to the strong deposition of PANI on CNCs. At a high charge density, the PEDOT is not deposited on the CNC surface but forms a gel-like film. The CNC/PEDOT displays reduced volumetric results during the charge-discharge reaction. In the case of CNC/PANI, PANI is strongly deposited on the CNC surface, and it forms a porous morphology at a higher deposition charge to support ion and electrolyte interface movement during the supercapacitor process [104]. 

A different approach is the fabrication of layered CNC/PPy/PVP electrodes as superior electrochemical supercapacitors [105]. The hydrophilic CNC surface is modified by the adsorption of polyvinylpyrrolidone (PVP), enabling the growth of the PPy polymer on the CNC surface. The conductive CNC/PPy/PVP composites display a smooth, uniform PPY coating and a higher capacitance than the CNC/PPy electrode. The CNC/PPy/PVP composite exhibits a C_s_ of 338.6 Fg^−1^ at 2 A g^−1^, with a retention rate of 87.3% after 2000 cycles. The higher capacitance and superior cyclic stability are attributed to the smooth and uniform deposition of PPy on the CNC surfaces, facilitating the charge transfer and diffusion between the electrode/electrolyte interface. The hydrogen bonding and the hydrophobic interaction of PPy and PVP provide a stable PPy coating layer on the CNC surface. A pseudo-3-layer structure of the CNC/PPy/PVP also plays a role to enhance the super capacitance [105]. 

## 12. Cellulose Nanocrystal-Conductive Hybrid Electrode

**Figure 8:**
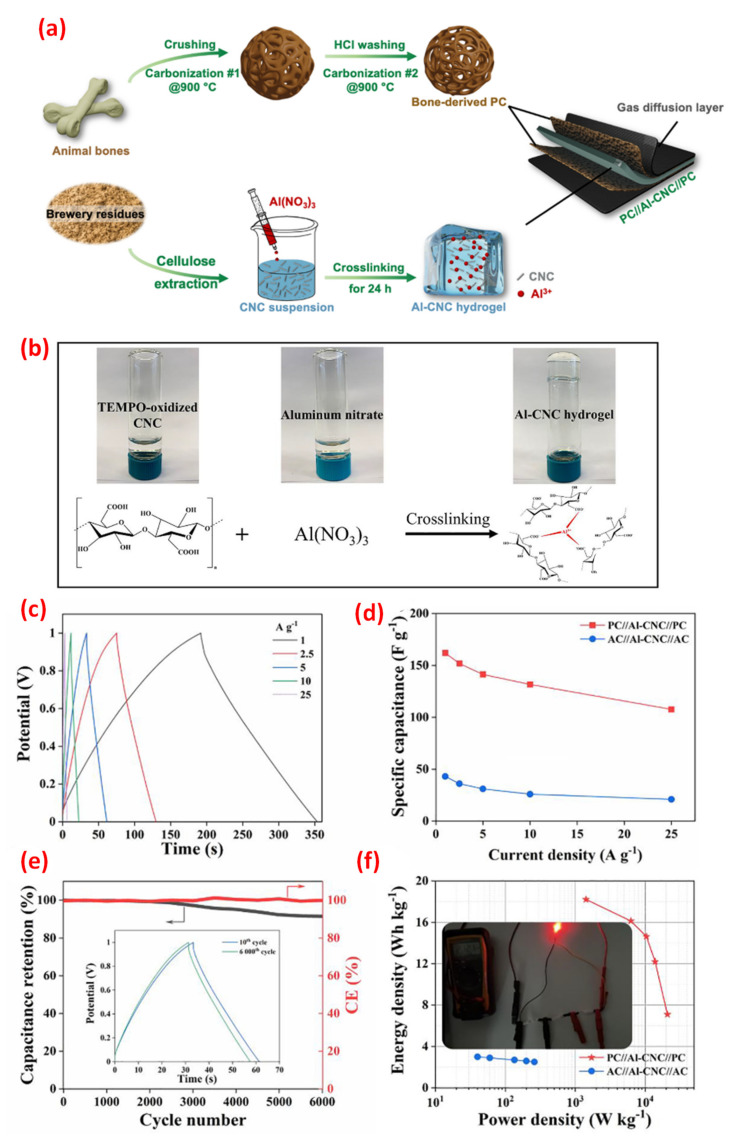
(**a**) The preparation of PC//Al−CNC//PC as a flexible supercapacitor, (**b**) The preparation of TEMPO mediated Al−CNC hydrogel, (**c**–**f**) The electrochemical properties, such as the GCD curve, specific capacitance, stability analysis and Ragone plot, of the PC//Al−CNC//PC electrode. Adapted with permission from [109]. Copyright 2022 Elsevier.

High conductive inorganic nanomaterials/CNC hybrids became very attractive for various potential applications owing to their unique size dependence and optical/catalytic properties [106]. Graphene oxide, transition metals, noble nanoparticles and metal oxides with high electrical conductivity are ideal materials for energy storage applications. The cellulose nanocrystals produce a strong interaction between the CNC surface and the inorganic nanomaterials via aggregation behavior. The dispersion of various nanoparticles on the CNC surface can develop new versatile nanomaterials for energy storage and environmental applications [107]. Carbon-based materials (graphene oxide, carbon nanotube (CNT), graphene, etc.) with outstanding conductive features are also chemically stable even under harsh environments. The deposition of carbon-based materials on the CNC surface results in lower conductivity due to the weak interaction and layer fall-off. The blending method is a suitable technique to trap the carbon-based nanomaterial inside the CNCs, which generates good electrical conductivity. Metal oxides exhibit higher electrical conductivity than graphitic particles; therefore, outstanding supercapacitor materials can be fabricated by combining them with CNCs. The metal oxide/CNC hybrid composites are prepared by coating, doping and co-precipitation methods [108]. Of note is the preparation of an Al-CNC transparent hydrogel electrolyte for supercapacitor applications [109]. CNCs are derived from brewery residues and are subjected to TEMPO (Figure 8). To obtain the transparent Al-CNC hydrogel, carboxylated CNCs are combined with high valence Al^3+^ via physical linking interaction. The Al-CNC hydrogel exhibits high electric conductivity, mechanical properties and optical properties. For comparison, Al^3+^ ion liquid electrolytes (LE) are prepared without CNCs. The Al-CNC electrolyte exhibits good ionic conductivity, high optical transmittance, excellent compression strength and tolerating properties to different angles (0° and 90°). The animal bone is carbonized at 900 °C to prepare porous carbon (PC) with a large surface area. An Al-CNC hydrogel-based system shows good electrochemical super capacitance and mechanical durability compared to the Al-LE system. The PC/Al-CNC electrode exhibits a specific capacitance of 804 Fg^−1^ at 1 Ag^−1^ compared to the 737 Fg^−1^ of the PC/Al-LE electrode. This result evinces that the 3D network of the transparent Al-CNC hydrogel electrolyte is attributed to enhanced specific capacitance [109]. 

**Figure 9:**
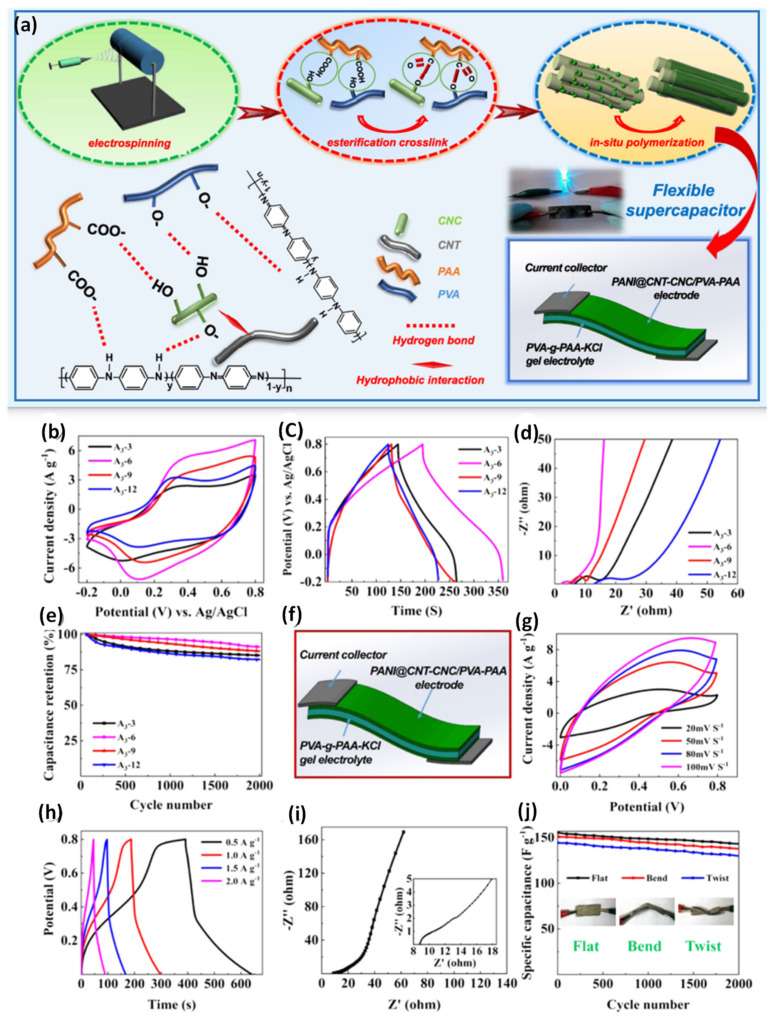
(**a**) The preparation PANI@ CNT−CNC/PVA−PAA; the electrochemical performance of the core-shell-structured PANI@CNT−CNC/PVA−PAA nanofibrous electrodes. (**b**) CV curves, (**c**) GCD curves, (**d**) EIS analysis and (**e**) cycling life of PANI@CNT−CNC/PVA−PAA. (**f**) The symmetrical flexible supercapacitor. (**g**) CV curves, (**h**) GCD curves, (**i**) Nyquist plots and (**j**) cycling life of the symmetrical PANI@ CNT−CNC/PVA−PAA flexible supercapacitor. Adapted with permission from [110]. Copyright 2019 American Chemical Society (ACS).

A high-performance and flexible PANI@CNT−CNC/PVA− PAA electrode (Figure 9) can be prepared by electrospinning and thermal treatment followed by a polymerization coating process for supercapacitor applications [110]. Poly(vinyl alcohol, PVA) and poly(acrylic acid, PAA) are utilized to prepare an electrospun nanofibrous membrane, and CNCs stabilized CNTs are used to enhance the mechanical and electrochemical functions. CNCs are obtained using sulfuric acid hydrolysis and ultrasonic treatment. During this step, negatively charged ions are formed to stabilize the CNTs on the CNC surface. To obtain the CNT−CNC/PVA−PAA membrane, a lab-scale electrospinning method is applied. The thermal treatment process triggers the esterification crosslinking reaction between the OH groups of CNCs and the –COOH groups of PAA. To enhance the electrochemical behavior of the CNT−CNC/PVA−PAA membrane, aniline is deposited and polymerized on the membrane to generate PANI@CNT−CNC/PVA−PAA. The specific capacitance of the PANI@ CNT−CNC/PVA−PAA is 164.6 Fg^−1^, which can be attributed to the synergetic effect between the CNC-CNT hybrid and PANI. The high porosity, the large surface area and the existence of PANI can enhance the capacitance of the prepared membrane. Particularly, a large surface area constructs enormous active sites for the faradaic reaction and increases the interaction between the electrode/electrolyte interface. The PVA–PAA polymeric matrix also promotes the electrolyte interface area to enlarge the redox reactions on the PANI. The developed electrode is considered a symmetrical supercapacitor by sandwiching the PVA–PAA–KCl electrolyte between the PANI@CNT−CNC/PVA−PAA electrodes. The symmetrical supercapacitor exhibited superior capacitance values of 155 Fg^−1^, with a retention rate of 92% after 2000 cycles. The calculated specific energy (E_s_) and specific power (P_s_) values are 13.8 Wh kg^−1^ and 200.3 W kg^−1^ for the PANI@CNT−CNC/PVA−PAA electrode [110]. 

A lightweight CNC–MWCNT–PPy electrode has been proven to be an efficient supercapacitor. CNCs are modified with NaIO_4_ and adipic acid dihydrazide to make aldehyde-modified CNCs (CNC–CHO) and hydrazide-modified CNCs (CNC–NHNH_2_), respectively. Sol-gel cross-linking enables the preparation of CNC aerogels from the CNC–CHO and CNC–NHNH_2_ with enhanced mechanical properties due to the chemical bonding between the amine and aldehyde groups. To facilitate the dispersion of MWCNTs on the CNC surface, the MWCNTs are suspended in a mixture consisting of sodium dodecyl sulfate (SDS) and taurocholic acid sodium salt [111]. The incorporation of these surfactants with MWCNTs enhances the reinforcing ability and high conductivity. The SDS and TCH (taurocholic acid sodium salt) surfactants help the dispersion of MWCNTs on the surface-functionalized CNC aerogels. The PPy conductive polymer is then deposited on the MWCNT/CNC aerogels by in situ polymerization of pyrrole using APS as an initiator. The lightweight CNC–MWCNT–PPy electrodes exhibited high super capacitance and a flexible nature along with a compressible nature. The dispersion and deposition of MWCNTs and PPy on the CNC surface result in 2.1 Fcm^−2^. 

**Figure 10:**
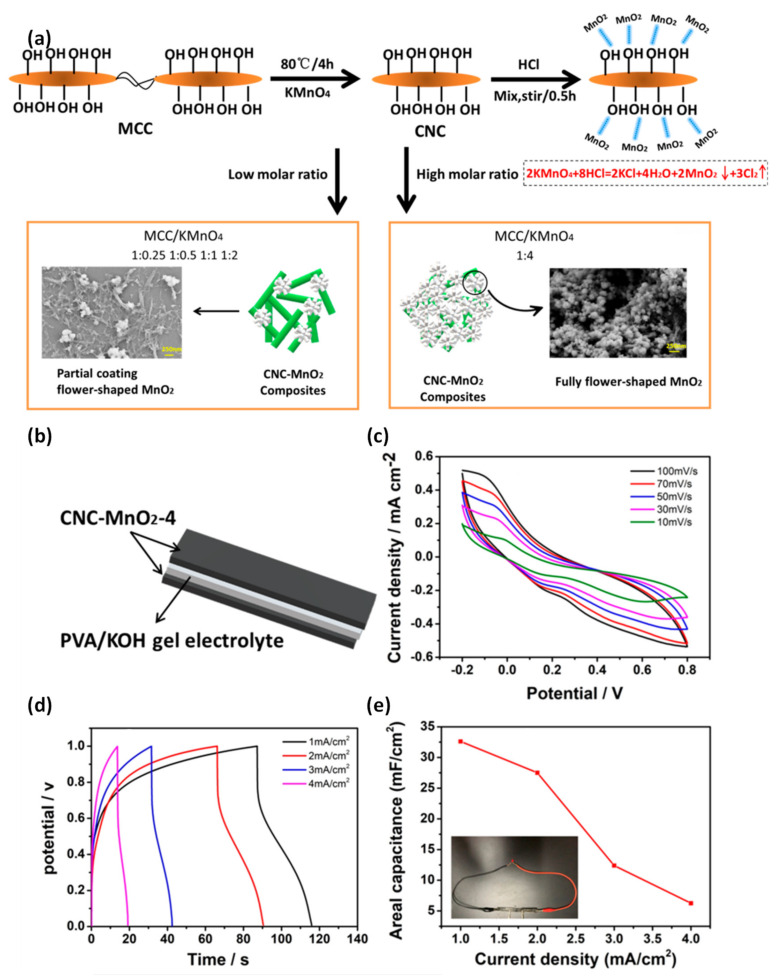
(**a**,**b**) Schematic synthetic route of the solid-state supercapacitor using the CNC−MnO_2_ electrode. (**c**–**e**) CV curves, GCD curves and the areal capacitance of CNC−MnO_2_. Adapted with permission from [112]. Copyright American Chemical Society (ACS).

An effective one-step hydrothermal preparation of CNC-MnO_2_ nanocomposites as solid-state electrochemical supercapacitors using commercial microcrystalline cellulose and KmnO_4_ has been reported [112]. A solid-state supercapacitor is assembled with sandwich typed CNC–MnO_2_ electrodes and the PVA/KOH electrolyte (Figure 10). The fabricated electrode exhibits a specific capacitance of 306.3 Fg^−1^, with good cyclic stability. The enhanced capacitance of the CNC–MnO_2_ reflects the large surface area of the MnO_2_ and CNCs with abundant hydrophilic hydroxyl groups. The CNC–MnO_2_ composite enhances the electrolyte uptake and transport through the pores and voids of the CNCs and MnO_2_, providing an excess ion diffusion pathway for the charge-discharge process. The energy density of CNC–MnO_2_ (42.59 Wh kg^−1^) is higher than that of pristine MnO_2_. The CNC–MnO_2_ is combined with the textile fabrics and conductive PPy to fabricate flexible electrodes for smart supercapacitor applications [112]. 

The synthesis of ternary PEDOT/CNC/MnO_2_ can be performed by one-step electropolymerization [113]. MnO_2_ is considered a good electrode material, but its conductivity is very modest, whereas PEDOT has mechanical flexibility and good electrical conductivity. However, the PEDOT/MnO_2_ composite still exhibits poor charge-discharge stability due to the effect of the continuous influx and outflow of electrolyte ions. To overcome this setback, CNCs are incorporated with PEDOT/MnO_2_ to improve super capacitance properties. A ternary PEDOT/CNC/MnO_2_ hybrid electrode exhibits a specific capacitance of 144.69 Fg^−1^ at 25 mVs^−1^ compared to PEDOT/CNC (63.57 Fg^−1^). The specific power (P_s_) and specific energy (E_s_) of PEDOT/CNC/MnO_2_ are 494.9 Wkg^−1^ and 10.3 Wg^−1^h, respectively. The PEDOT/MnO_2_ builds up the redox-active surface area, resulting in enhanced electrochemical capacitance [113].

## 13. Carbonized CNC Electrodes

High surface area porous carbon has often been synthesized from renewable and non-renewable resources such as waste-derived biomass, petroleum and coal through chemical activation and templating methods [114,115]. The fabrication of porous carbon from non-renewable resources is non-sustainable and very expensive. Highly efficient 3D porous carbon can be prepared from CNC using carbonization. During the carbonization process, non-conductive CNC is converted into conductive porous carbon materials. It is important to develop high-performance electrochemical supercapacitor materials without the influence of conductive polymers [116,117]. CNC-based carbon materials are prepared from CNCs with silica precursors by pyrolysis and etching. The fabricated carbon with a high surface area of 1400 m^2^/g possesses a C_s_ of 170 Fg^−1^ at 230 mAg^−1^ under the acidic electrolyte. The silica moiety triggers the generation of mesoporous carbon with a high surface area. The CNC-silica composite exhibits excellent super capacitance and good electrochemical retention stability [118]. 

A high surface area freestanding carbon film from CNCs and CNFs is created by the atomic layer deposition method [119]. Alumina is deposited on the CNC/CNF film at a low temperature and is carbonized at 900 °C for 2 h under the inner atmosphere. The Al_2_O_3_ layer helps to prevent CNC/CNF aggregation and preserve the fine structures of porous carbon during the carbonization process. The CNC/CNF film shows outstanding electrical conductivity and a surface area of 1200 m^2^g^−1^ compared to the traditionally prepared activated carbons. The specific capacitance of the carbonized CNF and CNC/CNF is 50 Fg^−1^ and 152 Fg^−1^, respectively. The interaction between the CNCs and CNFs augments the ion-transport efficiency, resulting in the higher performance of super capacitance activity in the CNC/CNF film. However, the specific capacitance is only 152 Fg^−1^, as the carbonized CNC/CNF film is interconnected with strong chemical interaction without any active materials with heteroatoms or functional groups [119]. 

The incorporation of heteroatoms (nitrogen) can be an effective approach to generating more active sites on the carbonized CNCs, which leads to the altering of the microstructure and properties of the CNCs [120]. An N-MFCNC nanocomposite can be prepared from melamine-formaldehyde functionalized CNC by one-step pyrolysis. The N-MFCNC electrode displayed a specific capacitance of 328 Fg^−1^ at 10 mVs^−1^ under acidic electrolyte, with cyclic stability of 95.4%. The existence of numerous nitrogen-active sites enhances the super capacitance activity of carbonized N-MFCNC [120].

**Figure 11:**
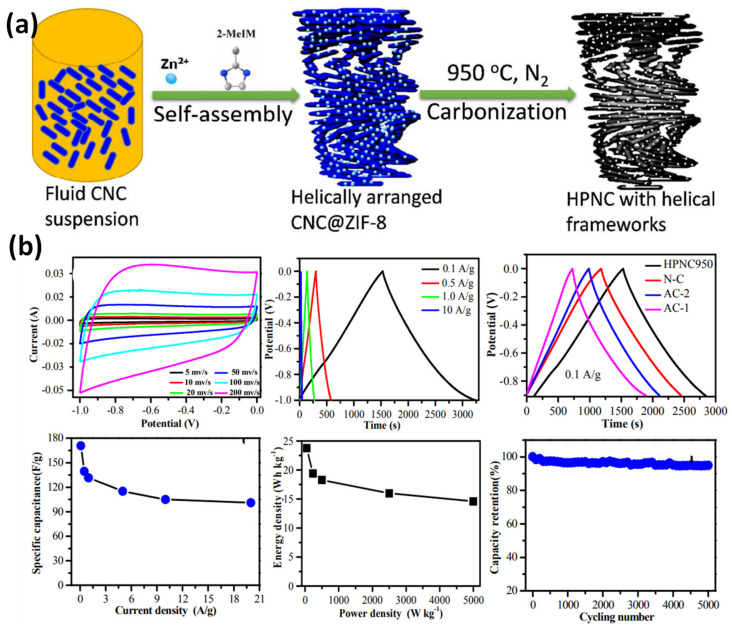
(**a**) Facile synthetic route of hierarchically porous N-doping carbon (HPNC), (**b**) Electrochemical characteristics of an HPNC composite in an aqueous KOH electrolyte. Adapted with permission from [121]. Copyright 2018 American Chemical Society (ACS).

A brief discussion is extended to the preparation of an N-doped highly porous carbon material [121] with a rod-like arrangement by the self-templated strategy from the in situ growth of ZIF-8 and CNCs followed by pyrolysis (Figure 11). The CNC structure is covered by micro and mesoporous ZIF-8-derived hollow carbon moiety without disturbing the chiral nematic phases of the CNCs. In addition, ZIF-8 helps to form a hierarchical porous structure and helically rod-like nanoparticles, enhancing the active sites and shortening the mass diffusion distance. Interestingly, CNCs are also involved in augmenting the surface area and pore size of the electrode materials. The CNC-ZIF-8-derived porous carbon exhibits 172 Fg^−1^ at 0.1 Ag^−1^, with long cyclic stability and a retention of 95% after 5000 cycles. The energy density (E_s_) and power density (P_s_) of N-doped carbon are 23.75 Whkg^−1^ and 50 W/kg^−1^, respectively. Such superior features of the N-doped carbon can be attributed to the conductive carbon rod-like helical structure and its porous nature, which facilitate fast electron migration in the electrode. The presence of nitrogen atoms increases the active sites and wettable nature of the electrode. In addition, the existence of macro, meso and micropores offers the spaces for storing electrolytes and rapid ion transportation and enhances the EDLC capacitance [121]. 

**Figure 12:**
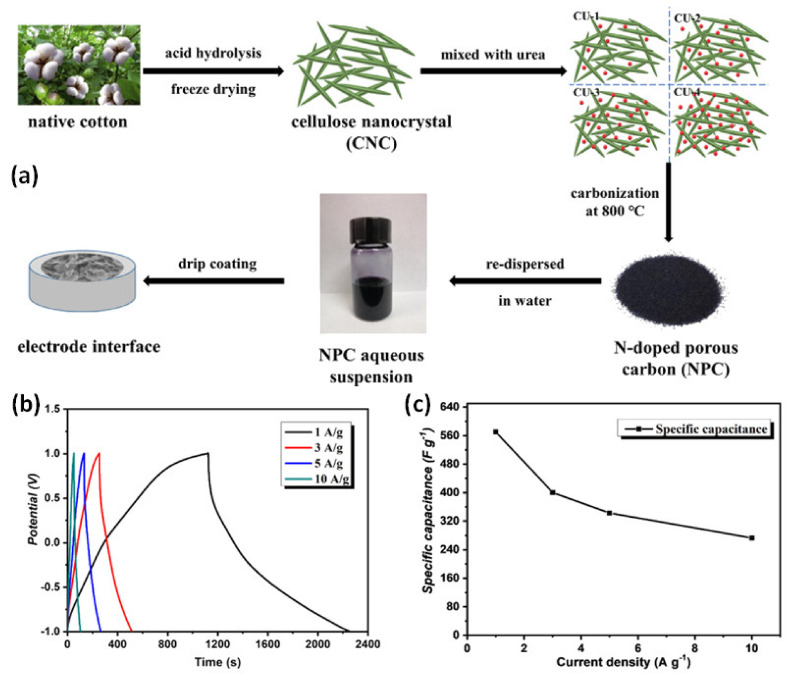
(**a**) N−doped cellulose nanocrystals (N−CNCs) are prepared from cotton and urea. (**b**,**c**) Electrochemical GCD curve and specific capacitance of N−CNCs under different current densities. Adapted with permission from [122]. Copyright 2020 Elsevier.

Of note is also the synthesis of N-doped porous carbon from a CNC suspension and water-soluble urea by carbonization for supercapacitor applications (Figure 12). Typically, the CNC surface is functionalized by acid hydrolysis to generate surface negative charges with sulfate groups. The synthesized N-doped carbon displays a surface area of 366.5 m^2^/g, a porous microstructure and improved electrochemical properties, with a capacitance retention of 91.2% after 1000 cycles. The N-doped carbon composite shows an electrochemical super capacitance of 570.6 Fg^−1^ at 1 Ag under the basic electrolyte. The result of the higher super capacitance and double layer capacitance characteristics of the N-doped carbon is due to the presence of nitrogen heteroatom in the composite. A symmetrical supercapacitor is fabricated with N-doped carbon materials for investigating practical super capacitance performance. The N-doped carbon electrode exhibits a specific capacity of 119 Fg^−1^, with an outstanding stability retention of 99.8% after 5000 cycles [122]. 

CNCs with high crystallinity due to the specific chemical composition and intermolecular interactions are considered advanced functional materials for various energy applications including supercapacitors and Li-ion batteries. The conversion of CNCs to porous carbon materials further enhances the supercapacitance properties. Renewable and low-cost CNCs are holding an incredible significance and are ideal candidates with an enormous potential for the development of supercapacitor electrodes. A comparison of the capacitance, power density and energy density of various CNC-based electrodes is depicted in Table 2. 

## 14. Conclusions and Outlook

In recent years, the continuous research interest regarding the fabrication of biodegradable and renewable materials owes to the necessity of a sustainable and productive environment as an alternative to fossil fuels. Renewable and innocuous cellulose nanocrystals have been widely utilized for advanced functional and environmentally friendly materials in supercapacitors, batteries and fuel cells due to the merits of their chemical composition, their strong intermolecular interaction and their high crystallinity. The focus of our review is on explaining the importance of CNCs as a sustainable natural resource and their applications as electrochemical supercapacitors. This review discusses the extraction, surface functionalization and morphological characteristics of CNCs to ensure the ability of CNCs to function in the energy storage field. The pretreatment step is always important and needed for the removal of unwanted chemical components such as lignin, pectin and other impurities. The surface functionalization process improves the dispersion ability of CNCs within both hydrophilic and hydrophobic polymer matrices. Promising CNCs are modified with various conductive polymers, conductive carbons and inorganic hybrids for developing new biodegradable green nanomaterials for the most significant energy systems. In addition, the key features for fabricating advanced electrode materials by converting non-conductive CNCs to conductive CNCs have been discussed. The optimization of the heteroatom doping strategy with sustainable and low-cost CNCs will achieve the market commercialization of conductive electronic and storage devices. Undoubtedly, some challenges are existing, and significant research challenges are required to solve the following issues for a better understanding of CNC characteristics and sustainable energy applications.

i. The need to fabricate hierarchical CNCs with an existing millimeter thickness and multi-scale porous microstructure. Hierarchical CNCs would become a great potential candidate for electrochemical systems.
ii. The control and optimization of the surface functionalization on the CNC will be crucial to making novel CNCs for both hydrophobic and hydrophilic matrices.
iii. Biosynthetic alternatives allow for the development of advanced high-performance CNC materials. The incorporation of biological molecules on the CNC surface by bacterial cellulose can make a better impact on their superior properties.
iv. There is still a large research gap in improving the performance of super capacitance, energy and power densities.
v. The construction of metal-free porous and heteroatom-doped CNC using a low cost and sustainable approach from abundant biomass is still needed.

This review fosters research endeavors to develop advanced manufacturing processes and characteristics of CNCs that reflect the ultimate value of their potential as supercapacitors. It also promotes the sustainable utilization of renewable biomass and biopolymer resources. Besides CNCs, carboxylated nanocrystalline chitin can be prepared by the hydrolysis and oxidation of chitin by ammonium persulfate [123]. Chitin is another abundant fibrous macromolecule that forms the major constituent in the exoskeleton of arthropods and fungal cell walls. Like CNCs, supercapacitors can be prepared from carboxylated nanocrystalline chitin, a subject of future endeavors. Like CNCs [124,125], the cytotoxicity of chitin nanocrystals [123] is very minimal, and this feature is a prerequisite for the production and application of nanomaterials en masse. 

Conducting PPY has been used with CNCs and other polymers toward the development of supercapacitors. However, this conducting polymer can be decorated with zinc vanadium oxide and used as a supercapacitor electrode material [126]. Other competitive techniques include using graphitic carbon nitride-doped copper–manganese alloy as an electrode material for energy storage [127]. Of interest is the fabrication of a lightweight, flexible self-charging power pack by the prudent integration of two paper-based high-performance triboelectric nanogenerators [128]. A novel two-dimensional donor-acceptor conjugated copolymer has been used as single-junction polymer solar cells with over 10% efficiency [129]. A review of the reliability of supercapacitors in energy storage applications is available elsewhere [130,131], and the discussion topics include the failure mechanisms, lifetime modeling and reliability-oriented design of SCs. 

## Figures and Tables

**Table 1:** Comparison of various pretreatment processes of CNCs.

Methods	Size (nm)	Advantages	Disadvantages
Acid hydrolysis	Diameter: 3–15 Length: 100–300	✓ Uniform ✓ High crystalline	High pollution
Enzymolysis	Diameter: 3–50 Length: 100–1800	✓ Fewer reagents ✓ Less pollution	Hard preparation Not uniform
Physical (mechanical, ultrasonic)	Diameter: 3–50 Length: 100–2000	✓ Large quantity ✓ Simple synthesis	Very large size Large energy consumption
Physical + green chemistry	Diameter: <20 Length: 100–500	✓ Maximum yield ✓ Less pollution	The preparation process is complex

**Table 2:** Comparison of CNC-based supercapacitor electrodes.

Composite	Fabrication	Electrolyte	Capacitance (F/g)	Power Density (Wh/kg)	Energy Density (Wkg^−1^)	Ref
PEDOT/NCC	Electrochemical deposition	1.0 M KCl	117.02	11.44	99.85	[101]
PPY/CNC	TEMPO/polymerization	0.5 M KCl	248	-	-	[102]
PPY/CNC	Electrochemical deposition	0.1 M KCl	256	-	-	[103]
PANI/CNC	Electrochemical deposition	1.0 M HCl	488	-	-	[104]
PEDOT/CNC	Electrochemical deposition	1.0 M HCl	69	-	-	[104]
PPY/PVP/CNC	Polymerization	0.5 M KCl	338.6	-	-	[105]
Biowaste-derived carbon	Carbonization	Al-CNC hydrogel	804	425	18.2	[109]
PANI@ CNT− CNC/PVA−PAA	Electrospinning/ Polymerization	1 M H_2_SO_4_	164.6	13.8	200.3	[110]
CNC-MWCNT-PPY	Polymerization	0.5 M Na_2_SO_4_	2.1 F cm^−2^	-	-	[111]
CNC-MnO_2_	Thermal synthesis	PVA/KOH	306.3	42.59	-	[112]
PEDOT/CNC /MnO_2_	Electro polymerization	1 M KCl	144.69	10.3	494.9	[113]
Porous carbon from CNC	Silica etching	1 M H_2_SO_4_	170	-	-	[118]
CNC/CNF	ALD	2 M KOH	152	-	-	[119]
N-CNC	Pyrolysis	1 M H_2_SO_4_	352	48.8	39.85	[120]
N-CNC	Pyrolysis	6 M KOH	172	23.75	70	[121]
N-CNC	pyrolysis	6 M KOH	570.6	23.75	50	[122]

## Data Availability

No new data were created or analyzed in this study. Data sharing is not applicable to this article.
